# Malnutrition and Frailty as Independent Predictors of Adverse Outcomes in Hospitalized Older Adults: A Prospective Single Center Study

**DOI:** 10.3390/medicina61081354

**Published:** 2025-07-26

**Authors:** Abdurrahman Sadıç, Zeynep Şahiner, Mert Eşme, Cafer Balcı, Burcu Balam Doğu, Mustafa Cankurtaran, Meltem Gülhan Halil

**Affiliations:** 1 Department of Internal Medicine, Hacettepe University Faculty of Medicine, Ankara 06230, Türkiye; 2 Division of Geriatrics, Department of Internal Medicine, Hacettepe University Faculty of Medicine, Ankara 06230, Türkiye

**Keywords:** mortality, rehospitalization, hospitalized patients, malnutrition, frailty, Clinical Frailty Scale, GLIM

## Abstract

*Background and Objectives:* Adverse clinical outcomes are associated with malnutrition and frailty, which are highly prevalent among hospitalized older patients. This study aimed to evaluate their predictive value for the duration of hospitalization, short-term survival, and rehospitalization of patients admitted to internal medicine wards. *Materials and Methods:* This prospective cohort study included 134 acutely ill patients aged ≥50 years who were hospitalized in an internal medicine department and evaluated within the first 48 h of admission. Nutritional status was evaluated using the Mini nutritional assessment–short form (MNA-SF), Nutritional Risk Screening 2002 (NRS-2002), and Global Leadership Initiative on Malnutrition (GLIM) criteria. Frailty was evaluated using the FRAIL scale and Clinical Frailty Scale (CFS). The primary outcomes were prolonged hospitalization (>10 days), mortality, and rehospitalization at 3 and 6 months post-discharge. *Results:* According to MNA-SF, 33.6% of patients were malnourished; 44% had nutritional risk per NRS-2002, and 44.8% were malnourished per GLIM. Frailty prevalence was 53.7% (FRAIL) and 59% (CFS). Malnutrition defined by all three scales (MNA-SF, NRS-2002, GLIM) was significantly associated with prolonged hospitalization (*p* = 0.043, 0.014, and 0.023, respectively), increased rehospitalization at both 3 months (*p* < 0.001) and 6 months (*p* < 0.001). Mortality was also significantly higher among malnourished patients. Higher CFS scores and low handgrip strength were additional predictors of adverse outcomes (*p* < 0.05). In multivariable analysis, GLIM-defined malnutrition and CFS remained independent predictors of rehospitalization and mortality. *Conclusions:* Frailty and malnutrition are highly prevalent and independently associated with prolonged hospital stay, short-term rehospitalization and mortality. Routine screening at admission may facilitate early identification and guide timely interventions to improve patient outcomes. These findings might guide hospital protocols in aging health systems and support the development of standardized geriatric care pathways.

## 1. Introduction

Malnutrition is a clinical condition characterized by insufficient intake or absorption of essential nutrients, resulting in impaired physical and cognitive functions [1]. Frailty, which is frequently observed in older adults, reflects diminished physiological reserves and resilience, making individuals more vulnerable to external stressors [2]. These two conditions frequently overlap in older individuals, and inadequate dietary intake remains a central, shared mechanism contributing to both [3].

Depending on the assessment method used, the prevalence of malnutrition in hospitalized patients ranges from 20% to 50%, but it often remains underrecognized and insufficiently addressed during inpatient care [4]. According to the SHARE study, frailty prevalence among older adults varies considerably, ranging from 6% to 44% across European countries [5].

Hospitalization for acute illness further increases the risk of developing or worsening malnutrition and frailty due to increased metabolic demands, systemic inflammation, and catabolic stress [6]. The interaction these conditions significantly worsens clinical outcomes, including prolonged hospital stays, higher readmission rates, and increased short-term mortality [7]. Concurrent screening for both is advised in hospitalized older adults due to their shared risk factors, clinical manifestations, and therapeutic approaches [8]. Although several studies have addressed frailty and malnutrition separately, few have simultaneously evaluated their predictive utility using both GLIM and CFS in internal medicine inpatients.

This study aimed to evaluate the prevalence and prognostic significance of malnutrition and frailty in patients aged 50 years and older admitted to an internal medicine ward with acute medical conditions. We investigated their associations with length of hospital stay, 6-month mortality, and 3- and 6-month post-discharge rehospitalization. These findings might be useful in identifying high-risk individuals who might profit from focused geriatric care interventions and early nutritional and functional support.

## 2. Methods

### 2.1. Study Population

**Figure 1:**
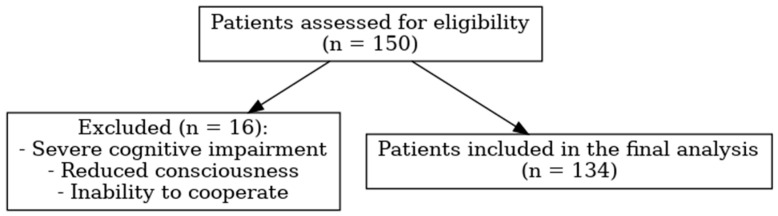
Flow diagram of patient selection and inclusion.

Between February and April 2023, this prospective cohort study initially screened 150 acutely ill patients aged ≥50 years who were admitted to the internal medicine department of a university hospital. The inclusion threshold of ≥50 years was chosen to reflect early clinical manifestations of frailty and malnutrition, which are increasingly observed in internal medicine patients below the age of 65. This approach aligns with previous studies evaluating age-related vulnerability in hospitalized populations [9]. After applying the exclusion criteria, 134 patients who met the eligibility requirements were included in the final analysis and were evaluated within the first 48 h of admission. Exclusion criteria included a history of diagnosed eating disorders, severe cognitive impairment that impaired cooperation or orientation, and reduced level of consciousness at the time of admission (Figure 1). 

### 2.2. Data Collection and Assessments

Baseline sociodemographic and clinical data were collected within the first 48 h of admission. These included age, sex, marital status, educational level, living arrangements, smoking and alcohol use history, comorbidities, reason for admission, history of nutritional support, history of falls or fractures within the past year, and relevant laboratory parameters. All primary variables used in the analysis were complete, with no missing data during collection.

### 2.3. Nutritional Assessment

Nutritional status was assessed using three validated instruments. For the Mini nutritional assessment–short form (MNA-SF), a score of ≥12 indicates normal nutritional status, 8–11 indicates risk of malnutrition, and ≤7 indicates malnutrition [10]. A score of ≥3 on the Nutritional Risk Screening 2002 (NRS-2002) was considered indicative of nutritional risk [11]. The Global Leadership Initiative on Malnutrition (GLIM) criteria were applied using a two-step diagnostic approach: first, a screening process was conducted, followed by the assessment of at least one phenotypic and one etiologic criterion. Based on GLIM definitions, malnutrition was then classified as either stage 1 (moderate) or stage 2 (severe) [12]. All nutritional assessments were conducted by the same researcher on the same day to ensure consistency.

### 2.4. Evaluation of Frailty

The FRAIL scale is a five-item screening tool used to evaluate fatigue, resistance, ambulation, illnesses, and weight loss. Patients are categorized as frail (3–5), pre-frail (1–2), or robust (0) based on their total score. The Turkish-validated version of the FRAIL Scale was used in this study [13]. The Clinical Frailty Scale (CFS) is a nine-point scale that ranges from 1 (very fit) to 9 (terminally ill). Patients were classified as frail (CFS > 4) or non-frail (CFS ≤ 4) [14].

### 2.5. Functional and Anthropometric Measurements

Standardized methods were followed to obtain anthropometric measurements. Height and weight were measured while the patient was standing at the time of admission. Body mass index (BMI) was calculated as weight in kilograms divided by the square of height in meters (kg/m^2^). Calf circumference was measured twice at the widest part of the non-dominant leg, and the average value was recorded. Handgrip strength (HGS) was assessed using a Takei handheld dynamometer to evaluate muscle strength. Taking three consecutive measurements from the dominant hand—unless medically contraindicated—the highest value was recorded. Low muscle strength was defined as <27 kg for men and <16 kg for women, according to the EWGSOP2 criteria.

### 2.6. Outcome Measures and Monitoring

Patients were prospectively followed up through telephone interviews conducted at 3 and 6 months after discharge. Outcome variables included length of hospital stay (categorized as ≤10 or >10 days), all-cause mortality, and rehospitalization at 3 and 6 months post-discharge. The 10-day threshold for prolonged hospitalization was based on previous geriatric research and signifies the clinical importance of prolonged inpatient admissions [9]. The median duration of hospitalization in our cohort was 11 days, supporting the appropriateness of this threshold.

### 2.7. Statistical Analysis

An a priori power analysis was conducted to determine whether the study could detect meaningful differences in key outcomes such as mortality and rehospitalization across patient subgroups. A sample size of 134 participants was sufficient to detect effect sizes as small as Cohen’s w = 0.24, indicating that the study was sufficiently powered to identify small-to-moderate differences in clinical outcomes, assuming a two-sided chi-square test with α = 0.05 and a desired power of 80%.

Data were analyzed using IBM SPSS Statistics, version 25. Descriptive statistics were reported as frequencies (%) for categorical variables, and as mean ± standard deviation or medians (min–max) depending on distribution. Normality of data was assessed using both visual methods (histograms, Q–Q plots) and analytical tests (Kolmogorov–Smirnov and Shapiro–Wilk).

Between-group comparisons were conducted using the chi-square or Fisher’s exact test for categorical variables, and Student’s *t*-test or Mann–Whitney U test for continuous variables, depending on data distribution. Threshold values used for risk stratification (e.g., MNA-SF ≤ 9, NRS-2002 ≥ 3, CFS > 4) were selected based on exploratory analyses within the cohort and their observed associations with clinical outcomes. These thresholds demonstrated the most distinct separation between high- and low-risk groups and were subsequently applied in multivariable and survival analyses. Multivariable logistic regression analysis was used to identify independent predictors of rehospitalization. Kaplan–Meier survival analysis and log-rank tests were applied to evaluate differences in overall survival. Covariates for multivariable models were chosen based on their univariate connection with outcomes at a significance level of *p* < 0.10 and their clinical relevance (e.g., age, sex, Charlson Comorbidity Index). The nutritional (MNA-SF, NRS-2002, GLIM) and frailty (FRAIL, CFS) factors were evaluated independently for prognostic significance in order to prevent collinearity.

A two-tailed *p*-value < 0.05 was considered statistically significant.

### 2.8. Ethical Statement

Ethics committee approval was received for this study from the ethics committee of our university with the decision number of GO 22/1259. Written consent was obtained from all participants. Constraints of the Declaration of Helsinki were adhered to during the study.

## 3. Results

A total of 134 patients were included in the study, with a mean age of 67.2 ± 10.4 years (range: 50–94); 75 (56%) were male and 59 (44%) were female. Comorbidities were present in 91.8% of patients (*n* = 123 out of 134). Hypertension (61.2%), diabetes mellitus (35.1%), cancer (32.1%), and coronary artery disease (32.1%) were the most prevalent comorbid conditions (Table 1). The primary reasons for hospitalization were pneumonia or pleural effusion (18.7%), intra-abdominal infections (18.7%), and acute kidney injury or electrolyte imbalances (20.9%).

Nutritional status was assessed using three validated tools. According to the Mini nutritional assessment–short form (MNA-SF), 50.7% of patients were at risk of malnutrition and 33.6% were classified as malnourished. A total of 44% of patients were found to be nutritionally at risk (score ≥3) according to the Nutrition Risk Screening 2002 (NRS-2002). Furthermore, 44.8% of patients satisfied the criteria for malnutrition according to the Global Leadership Initiative on Malnutrition (GLIM) criteria, with 29.9% classified as stage 1 and 14.9% as stage 2 (Table 2). Regarding frailty, 59% of patients were classified as frail according to the Clinical Frailty Scale (CFS), and 53.7% were identified as frail using the FRAIL scale (Table 2).

The median length of hospital stay was 11 days (range: 2–62). Among the patients, 68 (50.7%) experienced prolonged hospitalization (>10 days), while 66 (49.3%) were discharged within 10 days. Six percent (*n* = 8) of patients died within three months after discharge, and 46.2% (*n* = 62) were readmitted. The readmission and mortality rates at the 6-month follow-up were 48.5% (*n* = 65) and 11.2% (*n* = 15), respectively. There were no significant differences were observed in demographic variables, Charlson Comorbidity Index scores (*p* = 0.571), or cause of admission (infectious vs. non-infectious; *p* = 0.606) between patients with short (≤10 days) versus prolonged (>10 days) hospital stays. However, malnutrition was significantly more prevalent among patients with prolonged hospital stays. MNA-SF (42.6% vs. 24.2%, *p* = 0.043), NRS-2002 (62.7% vs. 41.3%, *p* = 0.014), and GLIM (54.4% vs. 34.8%, *p* = 0.023) all showed significantly higher proportions of malnourished patients in the >10-day group. In contrast, frailty prevalence did not differ significantly between groups when assessed by either the CFS (*p* = 0.085) or the FRAIL scale (*p* = 0.230) (Table 3).

At 3 months post-discharge, patients aged ≥65 years had significantly higher rehospitalization rates compared to those under 65 (60.8% vs. 41.7%, *p* = 0.027). At both 3 and 6 months, poor nutritional status as assessed by MNA-SF, NRS-2002, and GLIM was consistently associated with adverse outcomes, particularly rehospitalization (*p* < 0.001 for all). Furthermore, adverse outcomes at 3 months were significantly associated with lower handgrip strength (*p* = 0.003) and higher frailty scores (FRAIL: *p* = 0.027; CFS: *p* = 0.007). At 6 months post-discharge, both handgrip strength and CFS scores remained significantly associated with adverse clinical outcomes (Table 4).

In multivariable logistic regression models adjusted for age, sex, and the Charlson Comorbidity Index, several variables emerged as independent predictors of adverse outcomes. At 3 months, a lower MNA-SF score (OR: 0.717, *p* < 0.001), MNA-SF ≤ 9 (OR: 3.645, *p* < 0.001), NRS-2002 ≥ 3 (OR: 5.550, *p* < 0.001), GLIM-defined malnutrition (OR: 5.667, *p* < 0.001), and higher CFS scores (OR: 1.444, *p* = 0.019) were identified as independent predictors. At 6 months, the MNA-SF score (OR: 0.692, *p* < 0.001), NRS-2002 ≥ 3 (OR: 5.362, *p* < 0.001), and GLIM-defined malnutrition (OR: 6.071, *p* < 0.001) remained independent predictors of adverse clinical outcomes (Table 5).

**Figure 2:**
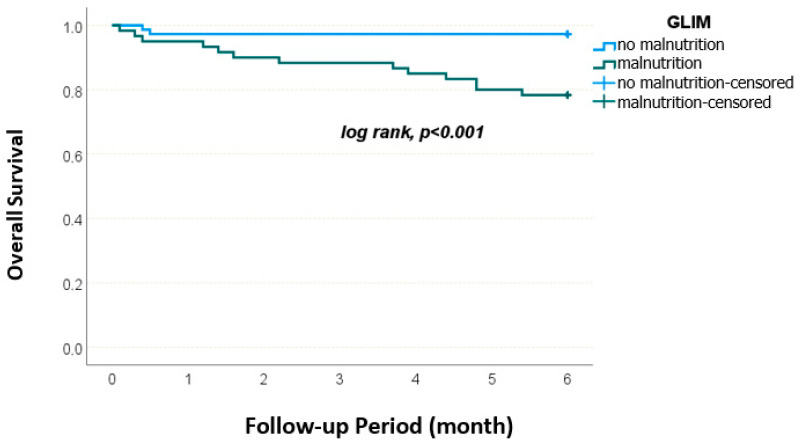
Kaplan–Meier survival curves stratified by nutritional status according to GLIM criteria. The 6-month survival rate was significantly lower in patients diagnosed with malnutrition compared to those without (*p* < 0.001).

**Figure 3:**
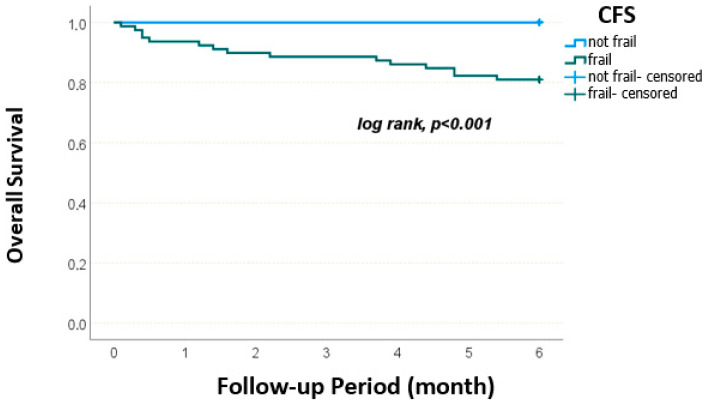
Kaplan–Meier survival curves stratified by frailty status according to the Clinical Frailty Scale (CFS). Frail patients had significantly lower survival compared to non-frail patients (*p* < 0.001).

The overall 6-month survival rate was 88.8% ± 2.7%. Survival rates differed significantly based on both nutritional and frailty status. According to the MNA-SF, patients with normal nutritional status had a 100% survival rate, while those at risk and malnourished had survival rates of 94.1% ± 2.9 and 75.6% ± 6.4, respectively (*p* = 0.002). According to NRS-2002, patients with scores <3 had a 6-month survival rate of 97.3% ± 1.9, whereas those with scores ≥3 had a rate of 78.0% ± 5.4 (*p* < 0.001). According to GLIM criteria, patients without malnutrition had a 6-month survival of 97.3% ± 1.9, whereas malnourished patients had 78.3% ± 5.3 (*p* < 0.001). Frailty status also significantly influenced survival (Figure 2). Patients with FRAIL scores of 0–2 had a 6-month survival of 96.8% ± 2.2, compared to those with scores of 3–5 had 81.9% ± 4.5 (*p* = 0.008). According to the Clinical Frailty Scale (CFS), non-frail patients had 100% survival rate, while frail patients had a significantly lower survival rate of 81.0% ± 4.4 (*p* < 0.001) (Figure 3).

## 4. Discussion

This prospective cohort study demonstrated that among acutely ill patients aged 50 years or older admitted to internal medicine wards, malnutrition and frailty were highly prevalent. We investigated using three validated nutritional assessment tools—MNA-SF, NRS-2002, and GLIM—malnutrition was independently associated with several adverse outcomes including prolonged hospital stay, increased 3- and 6-month rehospitalization rates, and higher short-term mortality. Particularly, when measured by the Clinical Frailty Scale (CFS), frailty was also an independent predictor of these adverse outcomes. These results highlight the need for methodical malnutrition and frailty screening at hospital admission in order to enable early targeted interventions, enhance risk stratification, and optimize discharge planning. Routine implementation of such screening measures could help establish more individualized and effective geriatric care pathways in internal medicine settings.

According to the MNA-SF, 50.7% of patients in our study were at risk of malnutrition, while 33.6% were classified as malnourished. The NRS-2002 (44%) and the GLIM criteria (44.8%) showed comparable prevalence rates, which highlight the significant undernutrition burden among hospitalized older adults. These findings are consistent with previous reports. For example, Balcı et al. reported malnutrition rates of 35.9% using GLIM and 38% with NRS-2002 in Turkish inpatients with acute medical conditions [15]. Although the GLIM criteria have been widely adopted in geriatric and oncologic populations, their application in general internal medicine is still limited. Our findings contribute to the growing body of evidence supporting its utility and prognostic significance in this larger population. Furthermore, the agreement among methods in this study suggests that, in cases of time or equipment limitations making comprehensive GLIM evaluation impractical, even fast screening instruments such as the MNA-SF may be clinically valuable.

Frailty was also highly prevalent in our cohort, with 59% of patients classified as frail by the Clinical Frailty Scale (CFS) and 53.7% by the FRAIL scale. These findings are consistent with previous studies on acutely hospitalized older adults, where, depending on the setting and assessment technique, frailty prevalence ranges from 40% to 60% [16,17]. In our study, the CFS proved particularly strongly prognostic, displaying both short- and medium-term rehospitalization as well as independent associations with 6-month mortality. Compared to the FRAIL scale, the CFS provides a more comprehensive evaluation by incorporating clinical judgment, functional capacity, and comorbidity burden. This multidimensional approach likely explains its better prognostic performance in our study. Furthermore, the CFS is a useful instrument for regular frailty screening in time-limited hospital settings since its viability and simplicity of use.

Malnutrition was strongly associated with prolonged hospital stay, particularly among patients with an MNA-SF score ≤9 and those at nutritional risk according to NRS-2002 (≥3). These findings are consistent with earlier multicenter studies from Brazil and Lebanon, which constantly reported that malnourished inpatients have longer hospital stays and more deprived in-hospital outcomes [18,19]. Significantly, our findings add to the increasing body of evidence confirming the prognostic relevance of the GLIM criteria in predicting not only long-term outcomes but also acute inpatient trajectories. While originally designed for nutritional diagnosis, GLIM consistently aligned with key outcome metrics in our cohort, reinforcing its utility in prognostic modeling and clinical decision-making. Notably, the predictive association observed for an MNA-SF cutoff of ≤9 suggests that nutritional risk may emerge earlier than traditionally assumed, highlighting a potential opportunity for earlier intervention. Considering the time and equipment constraints of most internal medicine wards, our integration of functional and anthropometric measurements—such as handgrip strength and calf circumference—into the GLIM framework provides an appropriate approach for bedside nutritional assessment. This change improves the tool’s feasibility without compromising its predictive validity, so increasing its fit for use in the real world. Further studies could confirm this simplified GLIM model as a possible standard for normal use in several clinical environments.

Beyond their diagnostic utility, each of the three nutritional tools—MNA-SF, NRS-2002, and GLIM—demonstrated predictive value for post-discharge outcomes, including 3- and 6-month rehospitalizations and 6-month mortality. These findings align with different international studies that confirm malnourished patients endure significantly worse post-discharge outcomes [20,21]. In addition to conventional nutritional indices, our analysis identified low handgrip strength and reduced calf circumference—clinical markers indicative of probable sarcopenia—as significant predictors of adverse events. Emerging data from large observational cohorts in China and Spain demonstrates that in older patients, muscle function and peripheral anthropometric indicators greatly affect recovery and readmission risk [22,23,24]. Including these functional criteria into standard nutritional evaluations could offer a complete approach for spotting high-risk patients who might profit from early physical and nutritional adjustments.

Although the CFS and FRAIL scale both were strongly associated with adverse outcomes at the 3-month follow-up, only the CFS kept predictive value at 6 months. This temporal difference corresponds with results of a South Korean cohort study showing better CFS performance in post-discharge readmission prediction [25]. Particularly in patients with cognitive impairment or communication difficulties, both common in older hospitalized populations, the FRAIL scale relies solely on self-reported information and dichotomous scoring, thus its sensitivity may be limited. In contrast, the CFS incorporates clinician judgment, physical function, and comorbidities, enabling a more nuanced stratification of risk. Our results offer more proof in favor of using the CFS as a strong and useful tool for internal medicine ward longitudinal frailty evaluation.

In multivariable logistic regression analyses adjusted for age, sex, and comorbidity burden, both malnutrition and frailty retained their status as independent predictors of adverse clinical outcomes. Among the three nutritional assessment instruments—MNA-SF, NRS-2002, and GLIM—each showed strong correlations with rising risk of 3- and 6-month rehospitalization, highlighting their clinical validity among older adults admitted to internal medicine departments who were acutely ill. In particular, GLIM-defined malnutrition generated some of the best odds ratios for adverse outcomes, underscoring its role not only in diagnosis but also in risk stratification. GLIM incorporates both phenotypic (e.g., weight loss, muscle mass reduction) and etiologic (e.g., inflammation or reduced intake) components, making it a comprehensive tool that reflects both chronic and acute nutritional deficits. On the other hand, the FRAIL scale lost statistical significance in adjusted models probably because of its lower sensitivity and conceptual overlap with the more thorough CFS. Notably, in adjusted analyses, the CFS kept its predictive ability, further supporting its role as a multidimensional tool for predicting clinical outcomes. Unlike screening-based tools like FRAIL, the CFS integrates physical performance, cognitive status, and functional capacity, which may explain its superior long-term prognostic utility. These findings highlight the additive prognostic importance of combining objective nutritional screening with clinician-rated frailty assessment at hospital admission.

Even though GLIM guidelines suggest using advanced modalities such as dual-energy X-ray absorptiometry (DEXA), bioelectrical impedance analysis (BIA), or computed tomography (CT) to assess muscle mass, routine internal medicine wards frequently lack access to these techniques. Calf circumference and handgrip strength were employed as practical surrogates for muscle mass and function in our investigation. Their predictive value—demonstrated both in our cohort and in prior studies [26,27]—supports their inclusion in clinical assessments, particularly in resource-limited environments where comprehensive body composition analysis is impractical.

### 4.1. Limitations

This study has several limitations. It was conducted at a single center with a relatively modest sample size, which may limit the generalizability of the findings. In addition, no in-hospital nutritional or rehabilitative interventions were implemented, which could have influenced post-discharge outcomes. Furthermore, particularly for people who are functionally impaired or chronically ill, the six-month follow-up period might not adequately depict the long-term consequences of malnutrition and frailty. Future multicenter studies with larger samples are warranted to validate and generalize our findings.

### 4.2. Strengths

Despite these limitations, the study has certain great strengths. Its methodological rigor is improved by its prospective design with organized follow-up and simultaneous use of several validated tools—including MNA-SF, NRS-2002, GLIM, CFS, and FRAIL. Furthermore, the study provides a reasonable model for nutritional and frailty screening in real-life internal medicine environments by including basic functional measurements including handgrip strength and calf circumference. Particularly, the finding of an MNA-SF cutoff of ≤9 as a major predictor of negative effects could offer a valuable insight for adjusting early intervention levels in clinical settings.

Taken together, our results highlight the clinical value of incorporating multidimensional and practical assessments of nutritional status and frailty—such as MNA-SF, GLIM, and CFS—into routine evaluation protocols for hospitalized older adults, so enabling timely risk stratification and targeted intervention. Furthermore, these findings may help guide the development of context-specific screening strategies applicable to the aging population across Europe.

## 5. Conclusions

Among hospitalized internal medicine patients, malnutrition and frailty are highly prevalent and are independently associated with short-term mortality, rehospitalization, and prolonged hospital stays. These findings underscore the importance of early and routine screening for frailty and nutritional status, especially in older adults who are admitted with acute illness. The Clinical Frailty Scale (CFS) and the Mini nutritional assessment–short form (MNA-SF) showed the best clinical viability and prognostic utility among the instruments that were evaluated. Notably, it was found that an MNA-SF cutoff score of ≤9 was independently predictive of adverse outcomes, indicating a potentially earlier threshold for nutritional intervention than previously understood. Systematic assessments of nutritional status and frailty upon hospital admission may enable prompt interventions, improve clinical outcomes, and reduce healthcare costs. Incorporating simplified GLIM and CFS tools in daily clinical practice may offer a feasible approach to improve outcomes in aging populations. To validate these findings and support evidence-based recommendations, more multicenter studies with larger cohorts and longer follow-up times are warranted.

These findings may also help integrate routine frailty and malnutrition screening in internal medicine wards and guide the development of hospital-wide geriatric care standards. In the context of Europe’s aging population, such strategies may enhance care coordination, promote more efficient resource allocation, and improve patient outcomes at the system level.

## Figures and Tables

**Table 1:** Baseline demographic and clinical characteristics of the study population (*n* = 134).

Parameters	Frequency (%), *n* = 134
Age, mean ± SD (range), years	67.2 ± 10,4
≥65 years	74 (55.2)
Sex, Male	75 (56)
Comorbidity (any)	123 (91.8)
Hypertension	82 (61.2)
Diabetes Mellitus	47 (35.1)
Malignancy	43 (32.1)
Coronary artery disease	43 (32.1)
Modified Charlson Index, median (min–max)	5 (2–10)
Low (≤3)	26 (19.4)
Middle (4–5)	47 (35.1)
High (≥6)	61 (45.5)

Variables were presented as n (%), mean ± SD or median (min–max).

**Table 2:** Demographic data of patients in terms of malnutrition, frailty, and associated parameters.

Parameters	Frequency (%), *n* = 134
Body Mass Index (BMI) mean ± SD, kg/m^2^	26.6 ± 5.1
Low BMI	13 (9.7)
Normal BMI	121 (90.3)
Calf circumference, mean ± SD, cm	32.6 ± 3.9
Nutrional Support, n (%)	8 (6)
Oral Nutrional Supplement (ONS)	6 (4.5)
Percutan Endoscopic Gastrostomia (PEG)	2 (1.5)
MNA-SF, median (min–max)	10 (3–14)
Normal	21 (15.7)
Risk	68 (50.7)
Malnutrition	45 (33.6)
NRS-2002, n (%)	
<3 score	75 (56)
≥3 score	59 (44)
GLIM, n (%)	
No Malnutrition	74 (55.2)
Malnutrition	60 (44.8)
Stage 1	40 (29.9)
Stage 2	20 (14.9)
FRAIL Scale, median (min–max)	3 (0–5)
0–2 score	62 (46.3)
3–5 score	72 (53.7)
Clinical Frality Scale (CFS), median (min–max)	4 (2–7)
Robust	55 (41)
Frail	79 (59)
History of Falls	33 (24.6)
Number of Falls in Last Year, median (min–max)	0 (0–6)
History of Fracture	13 (9.7)
Number of Fractures in Last Year, median (min–max)	0 (0–1)
Hand-Grip Strength, mean ± SD	25.8 ± 10.6
Low Hand-Grip Strength	88 (65.7)

Variables were presented as n (%), mean ± SD or median (min–max). BMI, body mass index; MNA-SF, Mini nutritional assessment–short-form; NRS-2002, Nutrition risk screening; GLIM, Global Leadership Initiative on Malnutrition; CFS, Clinical Frailty Scale.

**Table 3:** Comparison of malnutrition and frailty parameters according to hospitalization duration.

Parameters		Length of Stay, *n* (%)		*p* Value ^&^
		≤10 Days, *n* = 66	>10 Days, *n* = 68	
C-reactive protein (CRP), mg/L		0.9 (0.1–28)	1.25 (0.1–42)	0.044
BMI, mean ± SD, kg/m^2^		26.8 ± 4.7	26.5 ± 5.4	0.786
Calf Circumference, mean ± SD, cm		32.9 ± 4	32.2 ± 3.9	0.319
Nutritional Support, n (%)	No	65 (98.5)	61 (89.7)	0.062
	Yes (ONS/PEG)	1 (1.5)	7 (10.3)	
MNA-SF, median (min–max)	50–94 age	10 (4–14)	9 (3–13)	0.004
	<65 age	11 (4–12)	9 (3–12)	0.062
	≥65 age	10 (5–14)	7.5 (3–13)	0.023
MNA-SF, n (%)	Normal	14 (21.2)	7 (10.3)	0.043
	Risk	36 (54.6)	32 (47.1)	
	Malnutrition	16 (24.2)	29 (42.6)	
NRS-2002, n (%)	<3 score	44 (66.7)	31 (45.6)	0.014
	≥3 score	22 (33.3)	37 (54.4)	
GLIM, n (%)	No malnutrition	43 (65.2)	31 (45.6)	0.023
	Malnutrition	23 (34.8)	37 (54.4)	
	Stage 1	17 (73.9)	23 (62.2)	0.348
	Stage 2	6 (26.1)	14 (37.8)	
FRAIL Scale, n (%)	0–2 score	34 (51.5)	28 (41.2)	0.230
	3–5 score	32 (48.5)	40 (58.8)	
CFS, median (min–max)	50–94 age	4 (2–7)	5 (3–7)	0.007
	<65 age	3 (2–6)	3.5 (3–7)	0.168
	≥65 age	4 (3–7)	5.5 (3–7)	0.005
CFS, n (%)	Robust	32 (48.5)	23 (33.8)	0.085
	Frail	34 (51.5)	45 (66.2)	
History of Fall, n (%)		15 (22.7)	18 (26.5)	0.615
History of Fracture, n (%)		8 (12.1)	5 (7.4)	0.351
Low Hand-Grip Strength, n (%)		39 (59.1)	49 (72.1)	0.114

Variables were presented as n (%), mean ± SD or median (min–max). ^&^ Chi-square test for categorical variables, Student’s *t*-test for means, and Mann–Whitney U test for medians were used. MNA-SF, Mini nutritional assessment–short-form; NRS-2002, Nutrition Risk Screening; GLIM, Global Leadership Initiative on Malnutrition; CFS, Clinical Frailty Scale; BMI, body mass index.

**Table 4:** Comparison of malnutrition and frailty parameters with post-discharge rehospitalization.

Parameters		Rehospitalization in 3 Months			Rehospitalization in 6 Months		
		Yes, *n* = 70	No, *n* = 64	*p* Value ^&^	Yes, *n* = 80	No, *n* = 54	*p* Value ^&^
Male		43 (61.4)	32 (50)	0.183	51 (63.7)	24 (44.4)	0.027
≥65 age		45 (64.3)	29 (45.3)	0.027	50 (62.5)	24 (44.4)	0.039
Lymphocyte count, x103/mcl		1.2	1.6	<0.001	1.2	1.6	<0.001
		(0.1–34)	(0.1–25)		(0.1–34)	(0.1–4.5)	
Total protein, mean ± SS, g/dL		6.23 ± 0.78	6.54 ± 0.9	0.033	6.3 ± 0.9	6.5 ± 0.8	0.129
Albumin, mean ± SS, g/dL		3.3 ± 0.64	3.47 ± 0.55	0.115	3.2 ± 0.6	3.5 ± 0.5	0.007
Calf Circumference, mean ± SD, cm		31.3 ± 3.7	33.9 ± 3.8	<0.001	31.5 ± 3.7	34.1 ± 3.9	<0.001
MNA-SF, median (min–max)	50–94 age	7.5 (3–13)	10.5 (4–14)	<0.001	8 (3–13)	11 (4–14)	<0.001
	<65 age	8 (3–12)	11 (4–12)	0.008	9 (3–12)	11 (4–12)	0.014
	≥65 age	7 (3–13)	10 (6–14)	0.010	7.5 (3–13)	10 (6–14)	0.003
MNA-SF, n (%)	Normal	10 (14.3)	11 (17.2)	<0.001	11 (13.8)	10 (18.5)	<0.001
	Risk	25 (35.7)	43 (67.2)		31 (38.8)	37 (68.5)	
	Malnutrition	35 (50)	10 (15.6)		38 (47.5)	7 (13)	
NRS-2002, n (%)	<3 score	26 (37.1)	49 (76.6)	<0.001	33 (41.3)	47 (58.8)	<0.001
	≥3 score	44 (62.9)	15 (23.4)		42 (77.8)	12 (22.2)	
GLIM, n (%)	No malnutrition	25 (35.7)	49 (76.6)	<0.001	31 (38.8)	43 (79.6)	<0.001
	Malnutrition	45 (64.3)	15 (23.4)		49 (61.3)	11 (20.4)	
	Stage 1	27 (60)	13 (86.7)	0.058	31 (63.3)	9 (81.8)	0.307
	Stage 2	18 (40)	2 (13.3)		18 (36.7)	2 (18.2)	
FRAIL scale n (%)	0–2 score	26 (37.1)	36 (56.3)	0.027	32 (40)	30 (55.6)	0.076
	3–5 score	44 (62.9)	28 (43.8)		48 (60)	24 (44.4)	
CFS, median (min–max)	50–94 age	5 (3–7)	3 (2–7)	0.005	5 (3–7)	3 (2–7)	0.038
	<65 age	5 (3–7)	3 (2–6)	0.042	4.5 (3–7)	3 (2–6)	0.067
	≥65 age	5 (3–7)	4 (3–7)	0.169	5 (3–7)	4.5 (3–7)	0.549
CFS, n (%)	Robust	21 (30)	34 (53.1)	0.007	27 (33.8)	28 (51.9)	0.037
	Frail	49 (70)	30 (46.9)		53 (66.3)	26 (48.1)	
Low Hand-Grip Strength, n (%)		54 (77.1)	34 (53.1)	0.003	60 (75)	28 (51.9)	0.006
Modified Charlson Index, n (%)	Low (≤3)	10 (14.3)	16 (25)	0.082	10 (12.5)	16 (29.6)	0.010
	Middle (4–5)	22 (31.4)	25 (39.1)		26 (32.5)	21 (38.9)	
	High (≥6)	38 (54.3)	23 (35.9)		44 (55)	17 (31.5)	

Variables were presented as n (%), mean ± SD or median (min–max). ^&^ Chi-square test for categorical variables, Student’s *t*-test for means, and Mann–Whitney U test for medians were used. MNA-SF, Mini nutritional assessment–short-form; NRS-2002, Nutrition Risk Screening; GLIM, Global Leadership Initiative on Malnutrition; CFS, Clinical Frailty Scale.

**Table 5:** Independent effects of malnutrition and frailty parameters on post-discharge rehospitalization–multivariable logistic regression analysis.

Parameters	Rehospitalization in 3 Months		Rehospitalization in 6 Months	
	OR (95% CI)	*p*	OR (95% CI)	*p*
Age	1.032 (0.992–1.075)	0.120	1.022 (0.980–1.065)	0.320
Male	2.418 (1.076–5.434)	0.033	3.527 (1.481–8.399)	0.004
Modified Charlson Index	1.061 (0.862–1.308)	0.575	1.199 (0.958–1.501)	0.113
**MNA-SF score**	0.717 (0.604–0.850)	<0.001	0.692 (0.573–0.835)	<0.001
Age	1.027 (0.989–1.067)	0.164	1.015 (0.975–1.056)	0.465
Male	1.776 (0.835–3.778)	0.136	2.500 (1.131–5.528)	0.024
Modified Charlson Index	1.143 (0.940–1.389)	0.180	1.294 (1.039–1.611)	0.021
**MNA-SF ≤11**	1.489 (0.552–4.017)	0.432	2.017 (0.713–5.707)	0.186
Age	1.033 (0.993–1.075)	0.104	1.021 (0.980–1.064)	0.316
Male	2.103 (0.966–4.581)	0.061	2.936 (1.290–6.685)	0.010
Modified Charlson Index	1.052 (0.854–1.295)	0.634	1.193 (0.951–1.497)	0.127
**MNA-SF ≤9**	3.645 (1.686–7.882)	0.001	4.025 (1.767–9.169)	<0.001
Age	1.007 (0.967–1.049)	0.723	0.997 (0.955–1.040)	0.878
Male	2.073 (0.931–4.615)	0.074	2.805 (1.225–6.425)	0.015
Modified Charlson Index	1.089 (0.884–1.341)	0.424	1.253 (0.994–1.578)	0.056
**NRS-2002 ≥3**	5.550 (2.432–12.66)	<0.001	5.362 (2.233–12.87)	<0.001
Age	1.027 (0.987–1.070)	0.192	1.016 (0.975–1.060)	0.448
Male	1.955 (0.882–4.335)	0.099	2.742 (1.187–6.332)	0.018
Modified Charlson Index	1.054 (0.853–1.304)	0.626	1.206 (0.959–1.515)	0.109
**GLIM malnutrition**	5.667 (2.565–12.52)	<0.001	6.071 (2.572–14.33)	<0.001
Age	1.023 (0.985–1.063)	0.237	1.011 (0.972–1.053)	0.580
Male	1.834 (0.868–3.877)	0.112	2.300 (1.068–4.955)	0.033
Modified Charlson Index	1.096 (0.898–1.339)	0.368	1.255 (1.001–1.573)	0.049
**FRAIL scale 3–5 score**	1.938 (0.910–4.128)	0.086	1.563 (0.707–3.454)	0.270
Age	1.018 (0.979–1.058)	0.367	1.008 (0.968–1.049)	0.702
Male	2.098 (0.965–4.562)	0.061	2.510 (1.140–5.524)	0.022
Modified Charlson Index	1.069 (0.873–1.310)	0.520	1.232 (0.983–1.545)	0.071
**CFS**	1.444 (1.063–1.962)	0.019	1.284 (0.935–1.763)	0.122

OR: Odds Ratio, CI: Confidence Interval. MNA-SF, Mini nutritional assessment–short-form; NRS-2002, Nutrition Risk Screening; GLIM, Global Leadership Initiative on Malnutrition; CFS, Clinical Frailty Scale.

## Data Availability

The data presented in this study are available on reasonable request from the corresponding author. The data are not publicly available due to privacy and ethical restrictions related to patient confidentiality.
